# Being active with a total hip or knee prosthesis: a systematic review into physical activity and sports recommendations and interventions to improve physical activity behavior

**DOI:** 10.1186/s11556-022-00285-1

**Published:** 2022-02-28

**Authors:** Yvet Mooiweer, Martin Stevens, Inge van den Akker-Scheek, PAIR study group, Giuseppe Barone, Francesco Benvenuti, Mihai Berteanu, Laura Bragonzoni, Ileana Ciobanu, Dante Dallari, Ani Dimitrova, Ivo Dimitrov, Jorunn L. Helbostad, Alina Iliescu, Pasqualino Maietta Latessa, Andreea Marin, Alessandro Mazzotta, Ann-Katrin Stensdotter, Odd M. Hals, Håvard Østerås, Cristiano Paggetti, Erika Pinelli, Nataliya Shalamanova, Rumyana Shalamanova, Claudio Stefanelli, Matei Teodorescu, Nikolay Todorov, Nikolay Toselli, Maya Tsvetanova, Monica Unsgaard-Tøndel, Lora Yoncheva, Raffaele Zinno

**Affiliations:** Department of Orthopedics, University of Groningen, University Medical Center Groningen, P.O. BOX 30.001, 9700 RB Groningen, The Netherlands

**Keywords:** Total hip arthroplasty, Total knee arthroplasty, Physical activity, Sports, Sedentary behavior, Physical activity recommendations

## Abstract

**Objectives:**

Regular physical activity (PA) is considered important after total hip and knee arthroplasty (THA/TKA). Objective was to systematically assess literature on recommendations given by healthcare professionals to persons after THA and TKA and to provide an overview of existing interventions to stimulate PA and sports participation.

**Methods:**

A systematic review with a narrative synthesis including articles published between January 1995 and January 2021 reporting on recommendations and interventions. The PubMed, Embase, CINAHL and PsycInfo databases were systematically searched for original articles reporting on physical activity and sports recommendations given by healthcare professionals to persons after THA and TKA, and articles reporting on interventions/programs to stimulate a physically active lifestyle after rehabilitation or explicitly defined as part of the rehabilitation. Methodological quality was assessed with the Mixed Methods Appraisal Tool (MMAT). The review was registered in Prospero (PROSPERO:CRD42020178556).

**Results:**

Twenty-one articles reported on recommendations. Low-impact activities were allowed. Contact sports, most ball sports, and martial arts were not recommended. One study informed on whether health-enhancing PA recommendations were used to stimulate persons to become physically active. No studies included recommendations on sedentary behavior. Eleven studies reported on interventions. Interventions used guidance from a coach/physiotherapist; feedback on PA behavior from technology; and face-to-face, education, goal-setting, financial incentives and coaching/financial incentives combined, of which feedback and education seem to be most effective. For methodological quality, 18 out of 21 (86%) articles about recommendations and 7 out of 11 (64%) articles about interventions scored yes on more than half of the MMAT questions (0–5 score).

**Conclusion:**

There is general agreement on what kind of sports activities can be recommended by healthcare professionals like orthopedic surgeons and physiotherapists. No attention is given to amount of PA. The same is true for limiting sedentary behavior. The number of interventions is limited and diverse, so no conclusions can be drawn. Interventions including provision of feedback about PA, seem to be effective and feasible.

**Supplementary Information:**

The online version contains supplementary material available at 10.1186/s11556-022-00285-1.

## Background

Total hip arthroplasty (THA) and total knee arthroplasty (TKA) are clinically and cost-effective pain-relieving treatments for end stage osteoarthritis, and improve the ability to stay physically active [1]. In THA and TKA the original hip or knee joint is replaced by an artificial one. After either procedure it is of the utmost importance that persons maintain or adopt a physically active lifestyle) [2, 3]. Physical activity (PA) can be defined as any bodily movement produced by skeletal muscles that requires energy expenditure [4]. Regular PA is considered to be one of the most important lifestyle behaviors affecting health. It is proven to help prevent and treat noncommunicable diseases (NCDs) such as heart disease, stroke, diabetes, and breast and colon cancer. It also helps prevent hypertension, overweight and obesity, and can improve mental health, quality of life and well-being [5]. Being physically active on a regular basis also enhances fitness. Fitness is positively associated with functional autonomy in older adults [6]. Additionally, after THA and TKA individuals can benefit from being physically active as there are indications that this results in lower fall risk, increased bone density, improved prosthetic fixation and reduced risk of prosthetic loosening [2].

There are also negative consequences, one of the most important being prosthetic wear. The degree of prosthetic wear is not solely related to PA though. Both patient- and prosthesis-related factors contribute to the longevity of a prosthesis [7]. Moreover, the degree of wear depends not only on the amount of PA but also on the mechanical loading of the joint, which in turn depends on body weight, type of PA and technique (experienced or newbie athlete), where high-impact activity and poor motor control matter [8].

PA recommendations for persons after THA and TKA thus have to focus on amount and intensity of PA as well as on type of activity, including whether someone has experience with that activity. With respect to amount and intensity, the guidelines of the WHO published in 2020 can be used [9]. The most recent WHO guidelines recommend that every healthy adult (aged 18 to 65) do at least 150–300 min of moderate-intensity or at least 75–150 min of vigorous-intensity aerobic physical activity or an equivalent combination of moderate and vigorous activity throughout the week for substantial health benefits. Adults should also do muscle-strengthening activities at moderate or greater intensity that involve all major muscle groups on two or more days a week. For adults older than 65 it is recommended to add multicomponent physical activity that emphasizes functional balance and strength-training at moderate or greater intensity three or more days a week, to enhance functional capacity and to prevent falls. Lastly, it is recommended to limit the amount of time spent being sedentary. Although these recommendations are not specific for THA and TKA patients, they are also considered applicable to this patient group [2, 10].

For type of physical activity or sport after THA and TKA, a narrative review was published by Fawaz and Masri that gives an overview of activities allowed by healthcare professionals [11]. However, they did not systematically review the current literature so their overview might be missing information. The only overview of interventions or programs to enhance post-rehabilitation physical activity behavior of THA and TKA patients is that of Ishaku et al., who included papers up to November 2016. They concluded that studies showed a significant increase in time spent being physically active by participants in intervention groups compared to those in control groups [12]. However, research shows that a large group of patients remain inactive even when pain and functional deficits are gone after arthroplasty [10, 13–16]. Targeted interventions seem necessary to enhance physical activity behavior in this patient group.

The objective of this systematic review is therefore twofold: to systematically review the existing literature on recommendations given by healthcare professionals to patients after THA and TKA, and to provide an overview of existing interventions/programs described in the literature to stimulate a physically active lifestyle after THA and TKA.

## Methods

### Search strategy

A systematic review with a narrative synthesis was conducted. The review was registered in Prospero (PROSPERO: CRD42020178556) beforehand. A librarian of the Central Medical Library of UMCG was consulted for the search strategy. It was decided to perform one broad search strategy for both questions. The search strategy conducted is shown in Additional file 1: Appendix 1.

### Study selection

The PubMed, Embase, CINAHL and PsycInfo databases were systematically searched for original articles reporting on PA and sports recommendations given by healthcare professionals to persons after THA and TKA, and articles reporting on interventions/programs to stimulate a physically active lifestyle after rehabilitation or explicitly defined as part of the rehabilitation. Included persons had to be over 18 years of age. Articles written in a language other than English, review articles, case reports and study protocols were excluded. The search was conducted on 26 March 2020, and articles were searched from January 1995 onwards; an update of the search was done on 13 January 2021 following the guidelines of Bramer et al. [17].

Studies identified by the search strategy were imported to EndNote X9 (Clarivate Analytics Endnote X9.3.1, Philadelphia) and duplicates were removed following the guidelines proposed by Bramer et al. [18]. Articles were first screened for eligibility based on title and abstract. All articles extracted by the authors were screened for full-text eligibility. The screening procedure was performed by two authors (M.S. and I.A.S.) independently, and differences were solved by discussion. When needed, a third assessor (Y.M.) was consulted. Reference lists of included articles were screened for possible eligible articles that were missed in the initial search strategy.

**Fig. 1:**
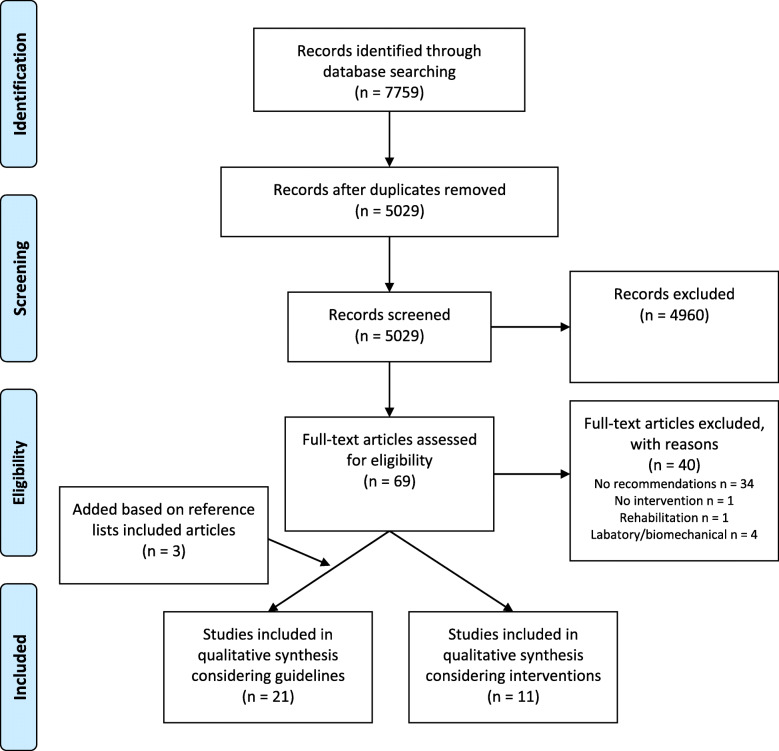
Meta-analysis of Observational Studies in Epidemiology (MOOSE) flowchart

The literature search yielded 7759 articles. After removing duplicates, 5029 unique articles remained. Subsequent reading of the titles and abstracts led to exclusion of 4960 articles. Full-text was assessed in the remaining 69. Of these, 18 articles reporting on recommendations were included. The reference lists of the included articles were manually checked and three additional articles were identified, making a total of 21. With respect to articles reporting on interventions, 11 were included after full-text screening. The entire procedure was performed by two authors (M.S. and I.A.S.). Once again, a third assessor (Y.M.) was consulted when needed (for flowchart, see Fig. 1).

### Data extraction and analysis

Data extraction was performed by two authors (M.S. and I.A.S.). For both research questions a separate table was created that included information about author and year, country, study design, sample size and characteristics, data collection period, type of sport/activity, measurement method and outcomes. Table 1 (first research question) includes the given recommendations and Table 2 (second research question) displays the intervention characteristics.

**Table 1:** Overview of characteristics and results of studies reporting on recommendations regarding physical activity after rehabilitation from total hip or knee arthroplasty

Author (year)	Arthroplasty	Study design	Sample size & characteristics	Data collection period (follow-up & time after surgery)	Type of (sports) activity	Measurement method	Outcome variables of interest	Recommendation
Amstutz and Le Duff [19] USA	THA	Survey	*N* = 661 Metal-on-metal hybrid HRA, female 30%, age 51.9 yrs. (14–78), BMI 26.5 (16.7–46.5)	Time after surgery: 10.1 [1–18] yrs	17 general sports activities	Questionnaire	Type, frequency & duration of sporting activities, Survivorship (revision for aseptic failure or wear), Impact & hip cycle scores	Return to sports is safe if treated with well-designed and well-implanted HRA.
Bradley, Moul [20] Great Britain	THA	Survey	*N* = 109 British Hip Society members	_	22 general sports activities	Web-based questionnaire	Level of impact (low, intermediate, high), Recommendation (allowed, allowed w experience, not allowed, undecided)	Low-impact sports allowed. Medium-impact sports, ± half of surgeons do not allow high-weight/low-repetition weight-lifting, ice-skating/roller blading. Rowing not allowed by minority of surgeons. High-impact and contact sports, road jogging, martial arts, high-impact aerobics not allowed.
Clifford and Mallon [21] USA	THA & TKA	Expert opinion	*N* = 2 Orthopedic surgeons	_	36/37 general sports activities	Consensus	Perceived impact (low, potentially low, intermediate, high)	Low-impact activities allowed, allowed w experience, medium-impact allowed w experience, high-impact not allowed.
Healy, Iorio [22] USA	THA & TKA	Literature review & survey	*N* = 54 Hip Society members, *N* = 58 Knee Society members	–	42 general sports activities	Questionnaire	Recommended/allowed, allowed w experience, no opinion, not recommended	Low-contact/impact sports activity recommended. High-contact/impact activity discouraged
Klein, Levine [23] USA	THA & TKA	Survey	*N* = 87 Hip Society members, *N* = 518 American Association of Hip & Knee Surgeons	_	37 general sports activities	Web-based questionnaire	Allowed, allowed w experience, not allowed, undecided	Low-impact activity allowed, medium-impact allowed or allowed w experience, high-impact not allowed
Laursen, Andersen [24] Denmark	THA & TKA	Survey	*N* = 45 Heads of orthopedic departments (performing ≥100 THAs or TKAs per year)	_	31 general sports activities	Questionnaire	Participate regardless of previous experience w activity, participate if person had experience w activity before surgery, do not participate in activity.	87% allow sports, 55% allow high-impact sports post-THA (35% if not experienced), 38% allow high-impact sports post-TKA (22% if not experienced)
McGrory, Stuart [25] USA	THA & TKA	Review & survey	*N* = 28 Mayo Clinic orthopedic surgeons, *N* = 13 consultants, *N* = 15 fellows or residents	_	28 general sports activities	Computerized literature search to identify citations pertaining to sports and prosthetic hip/knee surgery published between 1966 and 1993. Questionnaire	Recommended, not recommended, depends	No-impact/low-impact sports encouraged, high-impact prohibited. Results of survey in line with outcome of literature review
Meester, Wagenmakers [26] Nether-lands	THA & TKA	Survey	*N* = 117 Dutch Orthopaedic Association members (orthopedic surgeons)	–	40 general sports activities	Web-based survey, distinction made between ages < 65/> 65	Allowed, allowed w experience, discouraged, no advice. Knowledge about and application of international health-enhancing PA recommendations	Low-impact sports allowed. Most ball sports not recommended. Martial arts/contact/high-impact sports discouraged. Majority of surgeons discuss PA. Familiarity with PA recommendations is lacking.
Ollivier, Frey [27] France	THA	Matched case control study	*N* = 70 persons doing high-impact sports compared to *N* = 140 persons doing lower-impact activities	11 yrs. (10–15 yrs)	High-impact sports UCLA score 9–10 & low-impact sports UCLA score 1–4	HHS, HOOS, radiographic analysis (wear rate) and aseptic loosening/need for revision.	Function; dislocation rate; linear wear; survivorship (revision for mechanical failure/radiographic signs of aseptic loosening). Independent risk factors for failure.	Persons doing high-impact sports have better function than persons doing low-impact sports. High-impact sports can lead to mechanical failures.
Payo-Ollero, Alcalde [28] Spain	THA	Retrospective cohort study	*N* = 46, *n =* 13 female (58 hips) age 41 yrs. (37–48)	Average follow-up 7.5 years (1–11)	General sports activities	Telephone questionnaire	Sports recommended or advised against	Low-impact sports recommended (swimming, static biking, daily walking) Sports w high impact on hip not recommended. Contact sports allowed w previous experience.
Swanson, Schmalzried [29] USA	THA & TKA	Survey	*N* = 139 American Association for Hip and Knee Surgeons members (orthopedic surgeons)	–	15 general sports activities	Questionnaire	Unlimited, occasional [1–2 times/month], discouraged	Low-impact sports allowed. No consensus on medium-impact sports. High-impact sports discouraged. THA recommendations more liberal compared to TKA.
Thaler, Khosravi [30] Europe	THA	Survey	*N* = 150 European Hip Society members	–	47 general sports activities	Web-based questionnaire	Allowed, allowed if experienced, not allowed, no opinion. 4 time frames: within 6 weeks post-THA, 6–12 weeks post-THA, 3–6 months post-THA, more than 6 months post-THA.	Most physical activities were allowed 6 months post-THA. Experience in performing a distinct sport activity did not influence the recommendations to return to previous sports activities. Handball, soccer, football, basketball, full-contact sports, and martial arts not allowed.
Thaler, Khosravi [31] Europe	TKA	Survey	*N* = 120 European Knee Associates members (surgeons)	–	47 general sports activities	Web-based questionnaire	Allowed, allowed if experienced. Not allowed, no opinion. 4 time frames: within 6 weeks post-TKA, 6–12 weeks post-TKA, 3–6 months post-TKA. more than 6 months post-TKA.	Consensus for recommendation to allow 5 different sports in first 6 weeks, 7 sports at 6–12 weeks, 14 sports at 3–6 months, and 21 out of 47 activities 6 months postop. Number of sports recommended increases stepwise over postop time frames.
Vu-Han, Gwinner [32] Germany	TKA	Survey	N-101 German Arthroplasty Society members (surgeons)		30 general sports activities	Questionnaire	Recommendation: undecided, not recommended, w training, w.o. limitations	53.5% of surgeons recommend high-impact sports with adequate training, 36.6% do not recommend it at all, 5.9% recommend high-impact sports w.o. limitations. Most low-impact sports recommended after 3 months, while high-impact sports require at least 6 months of rehabilitation or rather not recommended at all.
Vu-Han, Hardt [33] Germany	THA	Survey	*N* = 99 German Arthroplasty Society members (surgeons)	–	30 general sports activities	Questionnaire	Recommendation: undecided, not recommended, w training, w.o. limitations	Low-impact sports recommended w.o. limitations and within 3 months post-THA. Return to high-impact sports advised by 51.5% of surgeons if the person received adequate training, 8.1% w.o. limitations, 34.3% did not recommend high-impact sports at all (3% left it up to the person). For high-impact sports, most experts recommended at least 6 months before return to sports. Basketball, boxing, soccer, gymnastics, handball, hockey, squash, climbing, volleyball, tennis and slope-skiing mostly not recommended or only w adequate training. Walking, swimming, hiking and level biking were activities the vast majority of surgeons recommended w.o. limitations or training. Recommendations seemed to vary for ballroom dancing, cross-country biking, bowling, dancing, e-scooters, fitness/weights, golf, horseback riding, jogging. Pilates, cross-country skiing, table tennis and yoga recommended w.o. limitations or w adequate training.
Witjes, Hoorntje [34] Nether lands	TKA & UKA	Survey	*N* = 82 Physiotherapists	–	32 general sports activities	Web-based questionnaire	Recommended, recommended w experience, possible but not recommended, impossible	Low-impact sports recommended. Medium/high-impact sports not recommended/considered impossible. More liberal in return to sports post-UKA than post-TKA.
Specific activities								
Gschwend, Frei [35] Switzerland	THA	Case control study	Group A: *N* = 50 regular alpine skiing and/or cross-country skiing, age 65 yrs. (47–84), weight 77 kg (44–100), height 1.73 m (148–193) Group B: *N =* 50, did no winter sports, age 65 yrs. (42–79), weight 78 kg (52–110), height 1.72 m (150–189)	10 yrs., measurements at 5 and 10 yrs	Alpine skiing and/or cross-country skiing	5-yr measurement: physical examination, questionnaire (hip, back, knee pain), radiographic examination (presence/location & extent of radiolucent lines, migration, tilting, subsidence). Rate of polyethylene wear (method Scheier et al. (1976)). 10-yr measurement: questionnaire/clinical/radiographic examination	Loosening & wear	Controlled alpine and/or cross-country skiing has no negative effect on acetabular or femoral component of hip replacements. Short-radius turns on steep slopes or moguls must be avoided.
Hara, Nakashima [36] Japan	THA	Laboratory study	*N =* 9 33% female, age 66 yrs. (55–84), BMI 25.0 kg/m^2^ (17.5–30.2)	Time after surgery: 4.8 [0.5–13.7] yrs	Golf	Kinematics	Hip kinematics during golf swing (hip movements, liner-to-neck contact & cup-head translation)	Golf is admissible due to dynamic hip stability.
Kloen, De Man [37] Netherlands	THA	Cohort study & literature review	*N =* 9 alpine skiers, 34% female, age 59.4 yrs. (47–70), weight 73 kg (52–95)	5.9 (1–13 yrs)	Alpine skiing	HHS, self-constructed questionnaire (downhill skiing-specific issues), radiographic analysis (weight-bearing AP/pelvic view, AP/lateral hip view)	Loosening, migration & wear	Downhill skiing is feasible, but ski with long turns on groomed slopes.
Mont, Rajadhaksha [38] USA	TKA	Survey	*N* = 33 (46 TKAs), United States Tennis Association high-level tennis players, 15% female, age 64 yrs. (30–79)	Time after surgery 7 yrs. (2–18)	High-level tennis	Questionnaire on clinical data of the TKA, general & sport-specific questions on tennis.	Surgeon’s advice on playing tennis. Years playing tennis, level, frequency, single/double. Stiffness and pain in mobility parameters (e.g. hitting, running, ground strokes, moving forward after serves to volley).	21% of surgeons approve playing tennis, 45% recommend only doubles, 55% oppose playing any tennis. High-level players were able to perform at preop level post-TKA. Players were satisfied with the TKA and ability to resume playing tennis.
Mont, LaPorte [39] USA	THA	Survey	*N =* 58 (65 THAs), United States Tennis Association players, 14% female, age 70 yrs. (47–89)	Time after surgery 8 yrs. (2–22)	Competitive tennis	Questionnaire on clinical data of the THA, general & sport-specific questions on tennis.	Surgeon’s advice on playing tennis. Years playing tennis, level, frequency, single/double. Stiffness and pain in mobility parameters (e.g. stroke by stroke, from follow-through to shifting weight into their stroke, mobility around the court).	14% of surgeons approve playing tennis, 34% recommend only doubles, 52% oppose playing any tennis. Players were extremely satisfied with their THA and their increased ability to participate in tennis. This select group of competitive players were able to perform at a better level post-THA than preoperatively.

AP = anterior posterior; BMI = body mass index; HHS=Harris Hip Score; HOOS=Hip Disability and Osteoarthritis Outcome Score; HRA = hip resurfacing arthroplasty; KG = kilogram; M = meters; *N =* number; THA = total hip arthroplasty; TKA = total knee arthroplasty; UCLA = University of California, Los Angeles; UKA = unicompartmental knee arthroplasty; w = with; w.o. = without; yrs. = years

**Table 2:** Overview of characteristics and results of studies reporting on interventions aiming to enhance physical activity behavior after THA/TKA

Author (year)	Arthroplasty	Study design	Sample size & characteristics	Inclusion criteria	Data collection period (follow-up & time after surgery)	Intervention	Type of sports	Measurement method*	Outcome variables of interest	Outcomes
Beck, Beyer [40] Germany	THA	RCT	*N* = 160 IG: *N =* 80, 52.5% female, median age 59.0 yrs. (51.1; 69.7), median BMI 26.4 kg/m^2^ (23.8; 28.6). CG: *N* = 80, 63.8% female, median age 61.9 yrs. (52.5; 70.0) median BMI 25.9 kg/m^2^ (23.7; 30.4).	General medical eligibility for hip rehab sports therapy, stable implant, age 18 yrs. or older.	Measurements at baseline, 6 and 12 months after surgery	IG: **rehabilitation sports therapy** program (endurance, strength, coordination, flexibility). CG: no rehab sports therapy.	General	Isokinetic dynamometry, postural stability, lactate threshold, WOMAC, HHS, pain (VAS), **UCLA scale**, EuroQol, EQ-5D.	Strength capacity not significantly better in IG. At one year IG subjects had less pain (WOMAC pain score (*p* = 0.023), size of effect small (r = 0.27). Health-related quality of life higher in intervention group at six months, size of effect small (*p* = 0.036, r = 0.25). The other parameters showed no significant changes differences. Median UCLA score was 7 in both groups at both six and twelve months.	No benefit of sports rehabilitation on functional outcomes compared to controls. Positive trends seen in some parameters. The unexpectedly high dropout rate had been underestimated in the planning of the trial.
Heiberg and Figved [41] Norway	THA	RCT	*N =* 60, mean age 70 yrs. (range 50–87) IG: *N =* 30, 70% female, education > 12 yrs. 57% CG: *N =* 30, 43% female, education > 12 yrs. 57%	Primary THA for OA and residence within an approximate 30-km radius from the hospital.	October 2008 to March 2010. Measurements preop and 3 & 5 months, 1 & 5 yrs. post-THA.	IG: a **supervised walking** skills-training program 3–5 months post-THA. 12 sessions, 70 min per session, 2x week. CG: not allowed to attend supervised physiotherapy during the same period, but encouraged to continue training on their own and to keep generally active.	Walking	6MWT, SCT, active hip ROM flexion/extension, 30-CST, HOOS, Self-efficacy (self-constructed), **UCLA activity scale**	IG and CG were equal on outcome measures of physical functioning, pain, and self-efficacy at 5 years (*p* > 0.05). In the total group, recovery course was unchanged from 1 to 5 years (p > 0.05), except for 9% improvement in ROM (*p* < 0.001) and increase in time on SCT of 18% (*p* = 0.004). Preop HOOS pain (*p* = 0.022) and HOOS sport (*p* = 0.019) predicted UCLA activity scale 5 years post-THA.	5 yrs. post-THA, the CG had caught up with the IG on physical functioning, participants led an active lifestyle. Those with worse preop scores on pain and physical functioning in sport were at risk of being less physically active in the long-term post-THA.
Hepperger, Gfoller [42] Austria	TKA	RCT (no blinded allocation)	*N* = 48 60% female, mean age 67 yrs. IG: *N* = 25 CG: *N* = 23	Persons post-TKA (55–75 yrs) 1–5 yrs. postop, committed to hiking 2–3 times/week over a 3-month period.	July–December 2015 Measurements prior to intervention period (pre-test), immediately after the 3-month intervention period (post-test) and 2 months after (retention-test).	IG: 3-month **guided hiking** program (2–3 times/week) CG: activities of daily living.	Hiking	SCT, KOOS, SF-36, extensor and flexor torque.	After hiking program, IG achieved faster overall walking times on the SCT. Time decreased from 4.3 ± 0.6 s (pre-test) to 3.6 ± 0.4 s (posttest) for the stair ascent (*p* = 0.060) and from 3.6 ± 0.6 s (pre-test) to 3.2 ± 0.5 s (post-test) for the stair descent (*p* = 0.036). IG showed significant improvement on KOOS subscales (symptoms/sport, recreation/QOL) from pre-test to retention-test (*p* < 0.01). No significant changes observed in IG. No effect on SF-36.	Results indicate moderate improvement in functional abilities and QoL of persons post-TKA who participated in a 3-month guided hiking program compared with CG subjects. Hiking did not have any acute detrimental effects on persons post-TKA during this study period.
Hoorntje, Witjes [43] The Netherlands	TKA	RCT	*N* = 97 58% female, mean age 58 yrs. (SD 4.8).	Persons < 65 yrs. suffering from debilitating knee OA and awaiting TKA, participating in a paid or voluntary job or working as an informal caregiver, and able to define and perform personal rehabilitation goals.	October 2015 to November 2017. Measurements preop and 6 months postop.	IG: Intervention using **GAS**. 3 personal activity goals: 1 ADL activity, 1 work activity, 1 leisure-time activity. CG: regular outpatient physical therapy	General	**Accelerometer**, Activ8. 5–7 consecutive days (24/7 in the month prior to TKA and 6 months post-TKA).	For the total group, a significant increase in PA of 9 min (±37) per day (*p =* 0.01) was observed and a significant decrease in sedentary time of 20 min (±79) per day (*p =* 0.02). No difference in standing time (*p* = 0.11). No difference CG and IG regarding changes in PA.	A small but significant increase in overall PA post-TKA, but no difference between GAS-based rehabilitation and standard rehabilitation was found.
Losina, Collins [44] USA	TKA	A factorial RCT	*N* = 202 57% female, mean age 65 yrs. (SD 8), 68% Bachelor degree, mean BMI 31 (SD 6) IG THC: *N* = 49 IG FI: *N* = 50 IG THC + FI: *N* = 52 CG: *N* = 51 Prior to TKR, participants walked a mean of 5032 steps/day (SD 2771). With the exception of step count, all characteristics are balanced across the arms.	Participants excluded if < 40 yrs., did not speak English, resided in nursing home, scheduled to undergo contralateral TKR or other surgery requiring hospitalization within 6 months, previously diagnosed with inflammatory arthritis or osteonecrosis affecting the knee, had a comorbidity that might prevent safe performance of moderate ambulatory PA, required a wheelchair or walker to ambulate preoperatively or did not have regular Internet access.	November 2013 through January 2016. Measurements preop and 6 months postop	4 groups: Attention control (CG), telephonic health **coaching** (THC), **financial incentives** (FI), THC + FI.	Walking	**Accelerometer** (Fitbit Zip), demographics, social and employment history, resource utilization, Knee injury Outcomes and Osteoarthritis Score (KOOS), EuroQol-5D (EQ-5D-3L), a general health Visual Analogue Scale (VAS), Risk Taking Index, Work Productivity and Activity Impairment questionnaire, **Yale Physical Activity Survey**, self-reported knee range of motion, components of the SF-36, MHI-5, Vitality Score.	Average daily step count at 6 months ranged from 5619 (SD 381) in THC arm to 7152 (SD 407) in THC + FI arm. Daily step count 6 months post-TKR increased by 680 (95% CI: − 94–1454) in control arm, 274 (95% CI: − 473–1021) in THC arm, 826 (95% CI: 89–1563) in FI arm, and 1808 (95% CI: 1010–2606) in THC + FI arm. PA increased by 14 (SD 10), 14 (SD 10), 16 (SD 10), and 39 (SD 11) minutes in the control, THC, FI, and THC + FI arms, respectively.	A dual THC + FI intervention led to substantial improvements in step count and PA post-TKR.
Paxton, Forster [45] USA	TKA	RCT	*N =* 45 IG: *N* = 22, female 50%, age 64 yrs. (SD 6), BMI 26.4 (SD 8.6) CG: *N* = 23, female 57%, age 63 yrs. (SD 7), BMI 29.9 (SD 10.7).	Participants 50–75 yrs. who underwent unilateral TKA	Initial assessments after completion of outpatient rehabilitation (6–8 weeks postop). Final assessments 12 weeks after beginning of intervention	IG: 12-wk program real time PA and **face-to-face feedback** CG: no PA feedback (current standard of care post-TKA)	General	Feasibility: retention, adherence, dose goal attainment, and responsiveness with pre- and post-intervention testing. PA: **accelerometer** (GT3X Actigraph Activity Monitor) Functional performance: TUG, 6-MWT, 4-MWT.	IG: 100% retention, 92% adherence (frequency of feedback use), and 65% dose goal attainment (frequency of meeting goals). IG average daily step count increased from 5754 (2714) (preop) to 6917 (3445) steps/day (postop).	The PA feedback intervention is a feasible intervention to use as an adjunct to conventional rehabilitation for persons with TKA and seems to be effective.
Piva, Almeida [46] USA	TKA	RCT	*N* = 44 IG: *N* = 22, female 82%, age 68.1 yrs. (SD 7.5), BMI 31.2 (SD 3.6) CG: *n* = 22, female 59%, age 68.3 yrs. (SD 5.5), BMI 29.3 (SD 4.1)	Participants > = 50 yrs., unilateral TKA 3–6 months before, no regular participation in exercise program	October 2011 to August 2013 6 months FU	IG: CBI program with exercise and **education** component. The education component of CBI to promote PA and healthy eating included two 30-min educational lectures during intervention week 1; mini-sessions of PA promotion were delivered in the subsequent weeks. CG: SCE. 3-month program followed by 3 months home exercise program (same for both groups)	Exercises	Feasibility of interventions assessed by adherence to supervised exercises, attrition and knee pain (WOMAC pain). Outcome measures: physical function (WOMAC PF, SF-36 PF, battery of performance-based tests) and PA using 7 days **accelerometry**.	Compared to the SCE group, the CBI group had less pain (*p* = 0.035) and better physical function based on the SF-36 (*p* = 0.017) and the single-leg stance test (*p* = 0.037). The other outcome measures did not demonstrate statistically significant differences between the two groups. Results from the responder analysis demonstrated that the CBI group had a 36% higher rate of responders in physical function than the SCE group. Also, the CBI group had 23% more responders in the combined domains of physical function and PA.	The CBI was found to be safe and well-tolerated, showing better outcome than the standard of care exercise program.
Pozzi, Madara [47] USA	THA	Case-series (*n* = 2)	N = 2 62 yrs., one female, one male Historical cohort as comparison (*N* = 32)	Persons 40–70 yrs., 3–9 months after unilateral THA	Measurements at baseline, end of intervention, 12 months post-THA	Exercise and **education** intervention, 18 supervised sessions over 6 weeks	Exercises	Feasibility and preliminary efficacy. HOS, hip abductor muscle strength, maximal voluntary isometric strength for quadriceps muscle, functional performance (TUG, SCT, 6-MWT, FSS), **IPAQ**, PSFS	Outcomes reported at individual level. Improved leg strength, weekly PA, and ability to perform demanding recreational and sports participation, without producing adverse effects. Feedback on the additional value of the health coach differed, leading to the conclusion that not all patients may benefit from this type of behavioral intervention.	This intervention could potentially increase activity levels and restore recreational participation in patients post-THA. Identifying those who may benefit from this intervention may help optimize outcomes without overusing resources.
Smith, Zucker-Levin [48] USA	TKA	RCT	*N* = 60 Female 65%, BMI 36.4 (SD 4.7) 10–18 months post-TKA IG: *N* = 30 CG: N = 30 Both groups: *N* = 24 ompleted final testing	Obese persons 1 year after unilateral TKA	Measurements at baseline, 8 weeks, end of intervention (16 weeks)	Both groups: 16-week tailored resistance and aerobic training designed to be completed at home with no supervision and minimal equipment based on ACSM guidelines for exercise prescription IG: exercise program and **fitness tracker** CG: exercise program only	Exercises	6-MWT, WOMAC, SF-36, ROM, knee extension strength.	Improvement on all outcome measures. The anecdotal reports from patients who received the fitness tracker technology indicated that many participants were engaged by the device and found it motivational (but no improvement in compliance with prescribed exercises).	The 16-week home-based exercise program is feasible and effective in improving strength and walk performance.
Trudelle-Jackson, Hines [49] USA	TKA	RCT	*N =* 13 Female 85%, age 63.5 yrs. (SD 7), BMI 34.8 (SD 7.6) IG: *N =* 7 CG: *N =* 6	Persons at least 6 months after primary unilateral or bilateral TKA, > 40 yrs	Pre- and post-test	IG: High-Velocity Training Exercises Plus **Step-Monitoring**, 8 weeks CG: **Step monitoring** only	Exercises	Muscle strength, muscle power, functional performance (6-MWT, SCPT), habitual walking behavior: number of steps/day along with minutes/week of moderate and/or vigorous PA (**pedometer**)	PA behavior: differences between pre-intervention and post-intervention values of PA behavior were not significant for minutes of MVPA (*p =* 0.09, r = −0.39) or for average daily steps (*p* = 0.09, r = 0.39) for the high-velocity training intervention group. The CG had significant improvement in number of daily steps (*p =* 0.01, r = 0.64), but not in minutes of MVPA (*p* = 0.38, r = 0.11).	No significant differences between IG and CG on amount of change in any of the outcomes. Based on these results, we could argue that providing a step-monitoring device like the simple pedometer used in this study or one of the many commercially available wearable technology may be more cost-effective than prescribing and monitoring a high-velocity training program.
Van der Walt, Salmon [50] Australia	TKA THA	RCT	*N* = 163 IG (FB): *N* = 81 CG (NFB): *N* = 82	Adults undergoing primary elective THA or TKA, 1 day postop	May–December 2016. Accelerometer measurements on days 1–14 postop, (PROMs) preop and 6 months postop.	FB group: **feedback** by means of accelerometer **on daily step goal**. NFB group: no feedback for 2 weeks postop and no daily step goal.	Walking	Garmin Vivofit 2 **accelerometer**, KOOS or HOOS,EuroQol-5D, satisfaction component of KSS, satisfaction with outcome of surgery, one-item question if they would have the same surgery again under the same circumstances	FB subjects had a significantly higher (*p* < 0.03) mean daily step count by 43% in week 1, 33% in week 2, 21% in week 6, and 17% at 6 months, compared with NFB. FB subjects were 1.7 times more likely to achieve a mean 7000 steps/day than NFB subjects at 6 weeks postop (*p* = .02). No significant difference in PROMs at 6 months. 90% of FB and 83% of NFB participants reported satisfaction with surgery results (*p* = 0.08). 6 months postop, 70% of subjects had a greater mean daily step count compared with their preop level.	The CBI program improves physical function and PA in patients several months post-TKA.

* Measure of physical activity in bold; ACSM = American College of Sports Medicine; ADL = activities of daily living; BMI = body mass index; CBI=Comprehensive Behavioral Intervention; CG = control group; CST = chair stand test; FB = feedback; FI = financial incentive; FSS = fatigue severity score; FU = follow-up; GAS = goal attainment scaling; HHS=Harris Hip Score; HOOS=Hip Disability and Osteoarthritis Outcome Score; HOS=Hip Outcome Score; IG = intervention group; IPAQ = International Physical Activity Questionnaire; KM = kilometer; KOOS=Knee Disability and Osteoarthritis Outcome Score; KSS=Knee Society Score; MHI-5 = Mental Health Inventory; 4-MWT = 4-min walk test; 6MWT = 6-min walk test; MVPA = moderate-to-vigorous physical activity; *N =* number; NFB = non-feedback; OA = osteoarthritis; PA = physical activity; PROMs = patient-reported outcome measures; PSFS=Patient-Specific Functional Scale; QOL = quality of life; RCT = randomized controlled trial; ROM = range of motion; SCE = standard of care exercise program; SCT = stair-climbing test; SCPT = stair climb power test; SD = standard deviation; SF-36 = Short Form 36; SF-36 PF=Short Form 36 Physical Functioning; THA = total hip arthroplasty; THC = Telephonic Health Coaching; TKA = total knee arthroplasty; TUG = Timed Up and Go Test; UCLA = University of California, Los Angeles; VAS=Visual Analog Scale; wk. = week; WOMAC=Western Ontario and McMaster Universities Osteoarthritis Index; WOMAC PF=Western Ontario and McMaster Universities Osteoarthritis Index Physical Functioning; yrs. = years

### Quality assessment

Quality assessment was performed using the Mixed Methods Appraisal Tool (MMAT) v. 2018 [51]. The MMAT is a critical appraisal tool designed to be used in reviews including qualitative, quantitative and mixed-method articles. For each of the five different study designs the MMAT comprises, it has five questions to determine whether the risk of bias on a certain aspect is low. If the risk of bias is low the question receives a “yes”, otherwise a “no”, and when it is not clearly described it receives a “can’t tell”. Since calculating a total score has been discouraged, it was chosen to present the ratings of the individual criteria [52]. The quality of the articles was judged by two researchers independently (M.S. and I.A.S.), and differences were solved by discussion, when needed with help of a third assessor (Y.M.).

## Results

### Recommendations for PA

#### Description of studies

Twenty-one studies [19–39] published between 1995 and 2021 were analyzed. Table 1 shows an overview of the study characteristics and results. The studies were conducted in Western countries (Western Europe and the United States), except for one study from Japan [36]. Most studies concerned the hip [19–30, 33, 35–37, 39], with fewer studies on the knee [21–26, 29–38].

#### Quality assessment

The quality of articles varied. The assessment of each article can be found in Additional file 2: Appendix 2A. Of the 21 articles included, none scored “yes” on all five questions of the MMAT, while 4 positive answers were given in five articles [23, 24, 27, 28, 35], 3 in thirteen articles [19, 20, 25, 26, 29–34, 36, 38, 39], 2 in one article [22], 1 in none of the articles, and none in two of the articles [21, 37]. There wasn’t a “no” score in ten articles [20, 23, 24, 27, 28, 30, 31, 34–36], while nine articles [19, 22, 26, 29, 32, 33, 37–39] received a “no” on one out of five questions. Further, one article [25] scored a “no” on two questions and 1 [21] on four questions. The remaining questions of the MMAT were assessed as a “can’t tell”.

#### Outcome

In sixteen studies [19–34] the focus was on general sports activities; in the majority of these studies self-constructed (web-based) questionnaires were used, distributed among orthopedic surgeons. One study [34] included physiotherapists. One study [27] focused on the influence of high-impact sports operationalized as a University of California, Los Angeles (UCLA) activity score of 9–10 versus low-intensity activities (UCLA score 1–4) on function, dislocation rate, linear wear and prosthetic survival. One study [26] informed on how far healthcare professionals use health-enhancing PA recommendations to stimulate persons after THA and TKA to become physically active again. No study included recommendations on sedentary behavior.

Five studies [35–39] focused on specific sports, two of which − including persons after THA − concerned alpine skiing and/or cross-country skiing [35, 37]. Focus was on the detrimental effect of skiing on loosening, migration and wear of the prosthesis. Two studies − one in persons after THA and one in persons after TKA − focused on tennis [38, 39]. Purpose was to characterize persons who play tennis after arthroplasty in terms of their functional abilities and degree of satisfaction. One study from Japan focused on playing golf [36] after THA. In a laboratory setting it was determined to what degree the golf swing had a detrimental effect on liner-to-neck contact and cup-head translation.

Overall, there is a general consensus on what kind of sports activities can or cannot be recommended.

### Interventions

#### Description of studies

In total 11 articles [40–50] were included describing an intervention or program that aims to enhance PA behavior during or after post-THA or post-TKA rehabilitation. Three studies [40, 41, 47] had post-THA participants, seven studies [42–46, 48, 49] post-TKA participants, and one study [50] aimed at both populations. All studies were RCTs, apart from one case series (*N* = 2 persons) [47]. The sample size of the RCTs ranged from 13 to 163 persons. The 11 articles studied 13 different interventions. Table 2 shows an overview of the study characteristics.

#### Quality assessment

The quality of articles varied. The assessment of each article can be found in Additional file 3: Appendix 2B. Of the 11 articles [40–50] included, two [41, 47] scored “yes” on all five questions of the MMAT, while 4 positive answers were given in two articles [46, 50], 3 in three articles [42, 45, 49], 2 in three articles [43, 44, 48], and 1 in one article [40]. There weren’t any “no” scores in three of the articles [41, 42, 47], while six articles [43, 45, 46, 48–50] received a “no” on one out of five questions. Two articles [40, 44] scored a ‘no’ on two questions. The remaining questions of the MMAT received a “can’t tell”.

#### Outcome

The 13 interventions to enhance PA were diverse: interventions using guidance from a coach/physiotherapist (*N* = 4) [40–43], interventions using technology-based feedback on PA behavior (*N* = 3) [48–50], face-to-face interventions (*N* = 1) [45], interventions including education on PA (*N* = 2) [46, 47], an intervention using goal-setting (*N =* 1) [43], an intervention using financial incentives (*N =* 1) [44], and an intervention using a combination of coaching and financial incentives (*N =* 1) [44]. To determine the effect of the intervention most studies (*N =* 6) used accelerometers to assess PA behavior [43–46, 49, 50]. The UCLA activity scale was used in two studies [40, 41], the International Physical Activity Questionnaire (IPAQ) in one [47]. Two studies did not use a measure of PA behavior [42, 48]. Moment of final follow-up assessment ranged from end of intervention to five years after intervention. Next to effectiveness the feasibility of five interventions was assessed [45–48, 50], which was considered good in all cases.

Combining the results from those studies using guidance (*N* = 4) [40–43], feedback (*N =* 4) [45, 48–50] and education (*N* = 2) [46, 47], feedback and education seem to be effective in enhancing PA behavior while guidance does not seem to enhance it.

## Discussion

The aim of this review was twofold: to provide an overview of PA and sports recommendations given by healthcare professionals and of existing interventions/programs to stimulate a physically active lifestyle after THA and TKA. For the first objective, 21 articles were found and in general it can be concluded that after both THA and TKA return to low-impact activities is allowed or recommended. Overall, contact sports, most ball sports (except for doubles tennis and table tennis), and sports in the martial arts category were not recommended. Interventions to enhance PA behavior were found in 11 articles, describing 13 interventions. Most interventions used guidance from a coach/therapist, with feedback about PA behavior or education as a means to enhance PA behavior, of which feedback and education seem to be the most effective.

Regarding the recommendations, the focus in the majority of the studies was on general sports activities. The number of general sports activities varied between 15 and 47, probably depending on what are considered general sports activities in the different countries. Most of the time self-constructed (web-based) questionnaires were used which were distributed among orthopedic surgeons and in one study among physiotherapists. Consensus statements were drawn based on the responses. Respondents were often members of national/international orthopedic associations or orthopedic staff at hospitals. In that sense, the outcomes and recommendations derived must be seen in the light of the PA and sports culture of the different countries, although overall it can be concluded that the line of the recommendations is more or less the same.

Contact sports and high-impact sports were discouraged: contact sports probably because of the high twisting forces as well as the large lateral and rearward forces on the joints that these activities entail [53], and high-impact sports are expected to increase wear rate and therefore negatively affect implant survivorship [27]. The UCLA score was often used to give an indication of the impact or intensity of sports activities, yet its suitability as a measure to determine intensity can be questioned: in our opinion it only gives a very rough indication. More research is needed, also with objective measurement methods, to gain more insight into the association between intensity of activity and implant survivorship.

The results of the studies (*n* = 5) that focused on recommendations for one specific sport were in line with the recommendations as described above, but highlighted the fact that preoperative experience with a specific sport matters. Two studies on persons after THA concerned alpine and cross-country skiing, and one golf and tennis. One study on persons after TKA concerned tennis. Especially when it comes to skiing it must be taken into account that cultural aspects too play a role, as residents of mountain regions will probably be more experienced. Tennis studies included competitive/high level players only, so results may not be representative of recreational tennis players.

With respect to applying the PA recommendations it can be concluded that only one study [26] informed the degree to which healthcare professionals use these recommendations to stimulate persons after THA and TKA to become physically active again. Although the WHO recommendations apply to the general population [9], these can also be used for persons after THA and TKA, while taking into account the pros and cons of different activities in relation to survival of the prosthesis. Not following the recommendations in usual care could be ascribed to lack of priority and knowledge. In a British study it was concluded that doctors do not pay much attention to discussing the role of PA with their patients. Contrary to tobacco use and alcohol consumption, doctors tend to under-prioritize physical inactivity [54]. Nonetheless, advising patients to meet PA recommendations is of the utmost importance, as regular PA has been indicated to improve overall health and fitness [5, 6]. While sedentary behavior is increasingly recognized as having a negative impact on health, and is added to the WHO 2020 recommendations, no studies included recommendations on limiting sedentary behavior [9].

Regarding the interventions to enhance PA behavior, the 11 studies found can be considered low. The interventions were all different in terms of content, which hampered conclusions about their effectiveness. This is in line with the results of the systematic review of Hawke et al., although they used stricter inclusion criteria [55]. On the other hand, in their review Ishaku et al. concluded that studies show a significant increase in time spent being physically active, although their number of included studies was also low [12]. Besides, not all studies in our review used PA behavior as outcome measure, they used Patient Reported Outcome Measures (PROMs) (pain, function, quality of life (QoL)) and outcome of physical functioning tests instead. In an attempt to combine our results, it seems that adding a feedback component has a positive effect on PA behavior. Most studies used feedback from a device reporting daily step count. This enables individuals to monitor their activity easily; feasibility was proven to be good. Supervised PA however does not seem to achieve better results for PA behavior. Education about a physically active lifestyle seems to enhance PA behavior, yet this conclusion is based on only two studies, one of which was a case study about only two persons. And it is not only short-term effects and feasibility of interventions which should be investigated: the long-term effect on PA is what really counts − an actual change in PA behavior. Of the included studies, the follow-up was mostly limited to a few months or even only post-intervention; only one study looked at the effect of an intervention 5 years later [41]. Overall, the only conclusion that can be drawn from this review is that more research is needed into the effectiveness of interventions aiming to enhance PA behavior in persons after THA and TKA, which is in line with the studies of Hawke et al. and Ishaku et al. [12, 55]. The lack of interventions seems to reflect the lack of attention to the long-term benefits of THA and TKA, i.e. the ability to adopt a physically active lifestyle without pain and functional limitations due to osteoarthritis.

## End conclusion

Based on the outcomes of this review it can be concluded that there is a general consensus on what kinds of sports activities can or cannot be allowed or recommended, which is primarily based on consensus studies. With respect to the number of publications on interventions aiming to enhance PA behavior after THA and TKA, it must be concluded that unsatisfactory attention is given to this topic. The number of interventions aiming to enhance PA behavior is very limited and reported interventions are diverse in terms of content, so no conclusions can be drawn. Interventions including the provision of feedback about PA seem to be effective and feasible, and it is recommended to further explore their working mechanism. The methodological quality of the included studies differed considerably. More high-quality studies are needed to support the current evidence, with special attention for long-term effects of interventions on change of PA behavior.

### Implications for practice

Orthopedic surgeons, physiotherapists and other healthcare professionals involved in the care of persons after arthroplasty can use the general consensus to advise persons on the kind of sports activities that are allowed or can be recommended. With respect to amount and intensity of physical activity, healthcare professionals should be encouraged to stimulate persons to comply with the WHO recommendations [9]. They should likewise give more attention to limiting sedentary behavior. Interventions using PA feedback are advised for this purpose. The recommendations issued by the WHO can be used for persons after THA and TKA, taking into account the pros and cons of different activities in relation to survival of the prosthesis.

## Supplementary Information

**Additional file 1: Appendix 1.** Search strategy.

**Additional file 2: Appendix 2A.** Quality assessment recommendations.

**Additional file 3: Appendix 2B.** Quality assessment interventions.

## Data Availability

The datasets used and/or analysed for the current study are available from the corresponding author upon reasonable request.
