# Correction to: Being active with a total hip or knee prosthesis: a systematic review into physical activity and sports recommendations and interventions to improve physical activity behavior

**DOI:** 10.1186/s11556-022-00292-2

**Published:** 2022-04-26

**Authors:** Yvet Mooiweer, Martin Stevens, Inge van den Akker-Scheek, PAIR study group, Giuseppe Barone, Francesco Benvenuti, Mihai Berteanu, Laura Bragonzoni, Ileana Ciobanu, Dante Dallari, Ani Dimitrova, Ivo Dimitrov, Jorunn L. Helbostad, Alina Iliescu, Pasqualino Maietta Latessa, Andreea Marin, Alessandro Mazzotta, Ann-Katrin Stensdotter, Odd M. Hals, Håvard Østerås, Cristiano Paggetti, Erika Pinelli, Nataliya Shalamanova, Rumyana Shalamanova, Claudio Stefanelli, Matei Teodorescu, Nikolay Todorov, Stefania Toselli, Maya Tsvetanova, Monica Unsgaard-Tøndel, Lora Yoncheva, Raffaele Zinno

**Affiliations:** Department of Orthopedics, University of Groningen, University Medical Center Groningen, P.O. BOX 30.001, 9700 RB Groningen, The Netherlands

**Correction to: Eur Rev Aging Phys Act 19, 7 (2022)**

**https://doi.org/10.1186/s11556-022-00285-1**

Following the publication of the original article [1] the authors reported that given name of one of the PAIR study group members Nikolay Toselli is not correct. The correct name is “Stefania Toselli”.

The original article [1] has been updated.
