# Supplementary material for: Being active with a total hip or knee prosthesis: a systematic review into physical activity and sports recommendations and interventions to improve physical activity behavior
Source: Eur Rev Aging Phys Act. 2022 Feb 28;19:7. doi: 10.1186/s11556-022-00285-1 (PMC8903715; doi:10.1186/s11556-022-00285-1)
Supplement: Supplementary file 2 — Additional file 2: Appendix 2A. Quality assessment recommendations. [file 11556_2022_285_MOESM2_ESM.docx]

# Appendix 2A: Quality assessment recommendations

| Author (year) | Category of study design | Q1 | Q2 | Q3 | Q4 | Q5 |
| --- | --- | --- | --- | --- | --- | --- |
| Amstutz and Le Duff [21] | Quantitative descriptive | Yes | Yes | No | Can’t tell | Yes |
| Bradley, Moul [22] | Quantitative descriptive | Yes | Can’t tell | Yes | Can’t tell | Yes |
| Clifford and Mallon [23] | Quantitative descriptive | No | No | Can’t tell | No | No |
| Healy, Iorio [24] | Quantitative descriptive | Can’t tell | Can’t tell | Yes | No | Yes |
| Klein, Levine [25] | Quantitative descriptive | Yes | Yes | Yes | Can’t tell | Yes |
| Laursen, Andersen [26] | Quantitative descriptive | Yes | Yes | Yes | Can’t tell | Yes |
| McGrory, Stuart [27] | Quantitative descriptive | Yes | No | Yes | No | Yes |
| Meester, Wagenmakers [28] | Quantitative descriptive | Yes | Can’t tell | Yes | No | Yes |
| Ollivier, Frey [29] | Quantitative non-randomized | Can’t tell | Yes | Yes | Yes | Yes |
| Payo-Ollero, Alcalde [30] | Quantitative descriptive | Yes | Can’t tell | Yes | Yes | Yes |
| Swanson, Schmalzried [31] | Quantitative descriptive | Yes | Can’t tell | Yes | No | Yes |
| Thaler, Khosravi [32] | Quantitative descriptive | Yes | Can’t tell | Yes | Can’t tell | Yes |
| Thaler, Khosravi [33] | Quantitative descriptive | Yes | Can’t tell | Yes | Can’t tell | Yes |
| Vu-Han, Gwinner [34] | Quantitative descriptive | Yes | Can’t tell | Yes | No | Yes |
| Vu-Han, Hardt [35] | Quantitative descriptive | Yes | Can’t tell | Yes | No | Yes |
| Witjes, Hoorntje [36] | Quantitative descriptive | Yes | Can’t tell | Yes | Can’t tell | Yes |
| Gschwend, Frei [37] | Quantitative non-randomized | Yes | Yes | Can’t tell | Yes | Yes |
| Hara, Nakashima [38] | Quantitative descriptive | Yes | Can’t tell | Yes | Can’t tell | Yes |
| Kloen, De Man [39] | Quantitative descriptive | No | Can’t tell | Can’t tell | Can’t tell | Can’t tell |
| Mont, Rajadhaksha [40] | Quantitative descriptive | Yes | Can’t tell | Yes | No | Yes |
| Mont, LaPorte [41] | Quantitative descriptive | Yes | Can’t tell | Yes | No | Yes |

**Quantitative non-randomized**: **Q1**. Were the participants representative of the target population? **Q2**. Were measurements appropriate regarding both outcome and intervention (or exposure)? **Q3**. Were there complete outcome data? **Q4**. Were the confounders accounted for in the design and analysis? **Q5**. During the study period, was the intervention administered (or did exposure occur) as intended? **Quantitative descriptive**: **Q1**. Was the sampling strategy relevant to address the research question? **Q2**. Was the sample representative of the target population? **Q3**. Were the measurements appropriate? **Q4**. Was the risk of nonresponse bias low? **Q5**. Was the statistical analysis appropriate to answer the research question?
