# Supplementary material for: Being active with a total hip or knee prosthesis: a systematic review into physical activity and sports recommendations and interventions to improve physical activity behavior
Source: Eur Rev Aging Phys Act. 2022 Feb 28;19:7. doi: 10.1186/s11556-022-00285-1 (PMC8903715; doi:10.1186/s11556-022-00285-1)
Supplement: Supplementary file 3 — Additional file 3: Appendix 2B. Quality assessment interventions. [file 11556_2022_285_MOESM3_ESM.docx]

Appendix 2B: Quality assessment interventions

| Author (year) | Category of study design | Q1 | Q2 | Q3 | Q4 | Q5 |
| --- | --- | --- | --- | --- | --- | --- |
| Beck, Beyer [42] | Quantitative RCT | No | Yes | No | Can’t tell | Can’t tell |
| Heiberg and Figved [43] | Quantitative RCT | Yes | Yes | Yes | Yes | Yes |
| Hepperger, Gfoller [44] | Quantitative RCT | Can’t tell | Yes | Yes | Can’t tell | Yes |
| Hoorntje, Witjes [45] | Quantitative RCT | Can’t tell | Yes | Yes | No | Can’t tell |
| Losina, Collins [46] | Quantitative RCT | Can’t tell | Yes | No | No | Yes |
| Paxton, Forster [47] | Quantitative RCT | Can’t tell | Yes | Yes | No | Yes |
| Piva, Almeida [48] | Quantitative RCT | Yes | No | Yes | Yes | Yes |
| Pozzi, Madara [49] | Quantitative descriptive | Yes | Yes | Yes | Yes | Yes |
| Smith, Zucker-Levin [50] | Quantitative RCT | Can’t tell | Yes | Yes | Can’t tell | No |
| Trudelle-Jackson, Hines [51] | Quantitative RCT | Yes | Yes | No | Can’t tell | Yes |
| Van der Walt, Salmon [52] | Quantitative RCT | Yes | Yes | Yes | No | Yes |

RCT=randomized controlled trial. **Quantitative randomized controlled**: **Q1**. Was randomization appropriately performed? **Q2**. Were the groups comparable at baseline? **Q3**. Were there complete outcome data? **Q4**. Were outcome assessors blinded to the intervention provided? **Q5**. Did the participants adhere to the assigned intervention?
